# Management of Herpes Zoster Infection Using a Combination of Allopathic and Herbal Medication: A Case Report

**DOI:** 10.7759/cureus.55705

**Published:** 2024-03-07

**Authors:** Rosalyn Lalremtluangi, Suwarna Dangore-Khasbage, Rahul R Bhowate

**Affiliations:** 1 Oral Medicine and Radiology, Datta Meghe Institute of Higher Education and Research, Wardha, IND

**Keywords:** infection, herbal, herpes zoster, dermatome, allopathic

## Abstract

Herpes zoster (HZ) also known as shingles is the reactivation of varicella-zoster virus (VZV) that causes chickenpox. It usually occurs in adults after remaining dormant in the dorsal root ganglia or ganglia of the cranial nerves for several years. It typically manifests as vesicle crops in a dermatomal or "zosteriform" pattern, which shows vesicle, ulcer, and scab distribution that is unilateral, clustered, and linear in a dermatome that is supplied by a single nerve. Patients usually experience prodromal symptoms of deep, severe aching or burning pain. Medicinal treatment frequently includes antiviral drugs to decrease the severity of the lesion and steroidal drugs to reduce symptoms of inflammation. It is also a known fact that steroid has several adverse effects on patients due to which therapeutic drugs with lesser side effects may be given to patients such as herbal medications. This case presentation reports a patient with HZ viral infection who was successfully treated with a meticulous combination of conventional allopathic drugs with ayurvedic medication with a significant positive response to the medication.

## Introduction

Herpes zoster (HZ) is an infection that occurs due to the reactivation of varicella-zoster virus (VZV) [1]. After a person has contracted chickenpox, the virus remains latent in the dorsal root ganglia or ganglia in the cranial nerves and can reboot later in life, leading to HZ [2]. The reactivation of the virus typically occurs in individuals whose immune system is weak, in advanced age, or stressed [3]. It is characterized by painful, blisters that usually appear in a particular pattern along a single dermatome, which is an area of skin supplied by a single nerve. It is important to note that HZ is not a new infection but rather a reactivation of the dormant virus [3]. The condition may not be directly contagious, but the virus can be spread to a person who have not had chickenpox or varicella vaccine. This contact can lead to the development of chickenpox, not HZ [4]. A study done by Indian author revealed a higher occurrence of HZ in younger population of 31-40 years (24%) and 21-30 years (19%) [5].

Management of the lesions caused by varicella infection is mainly focused toward pain control (particularly to prevent the lesion from causing postherpetic neuralgia) hydration, supportive care, and definitive treatment to reduce the risk for dissemination, specifically in immunocompromised patients [6]. Medication such as acyclovir reduces infectivity and severity of the lesions. Corticosteroids are also used to reduce inflammation and to alleviate symptoms caused by the lesion [7]. Apart from this conventional medication given for HZ infection, the use of certain herbal products has also been reported to be effective for the management of HZ infection [8]. As herbal medications are known to cause less adverse effects as compared to allopathic medication, its use in the management of certain lesions has been entertained and supported [9]. This is a case report of a 36-year-old male with HZ infection where modification of management has been done using a combination of conventional drugs and herbal medications.

## Case presentation

**Figure 1:**
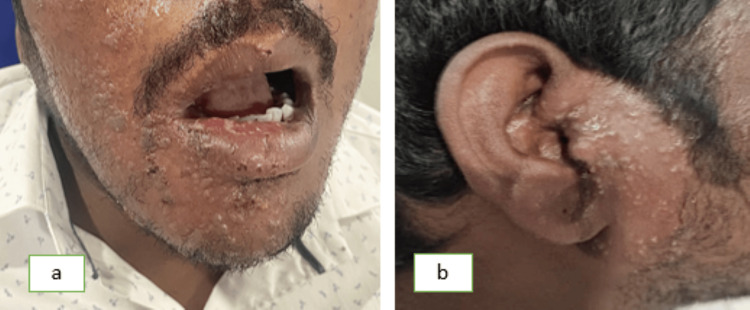
(a) Shows unilateral distribution of vesicles, ulcers, and scabs with erythema on the right region of jaw; (b) shows vesicles on the right auricular region

**Figure 2:**
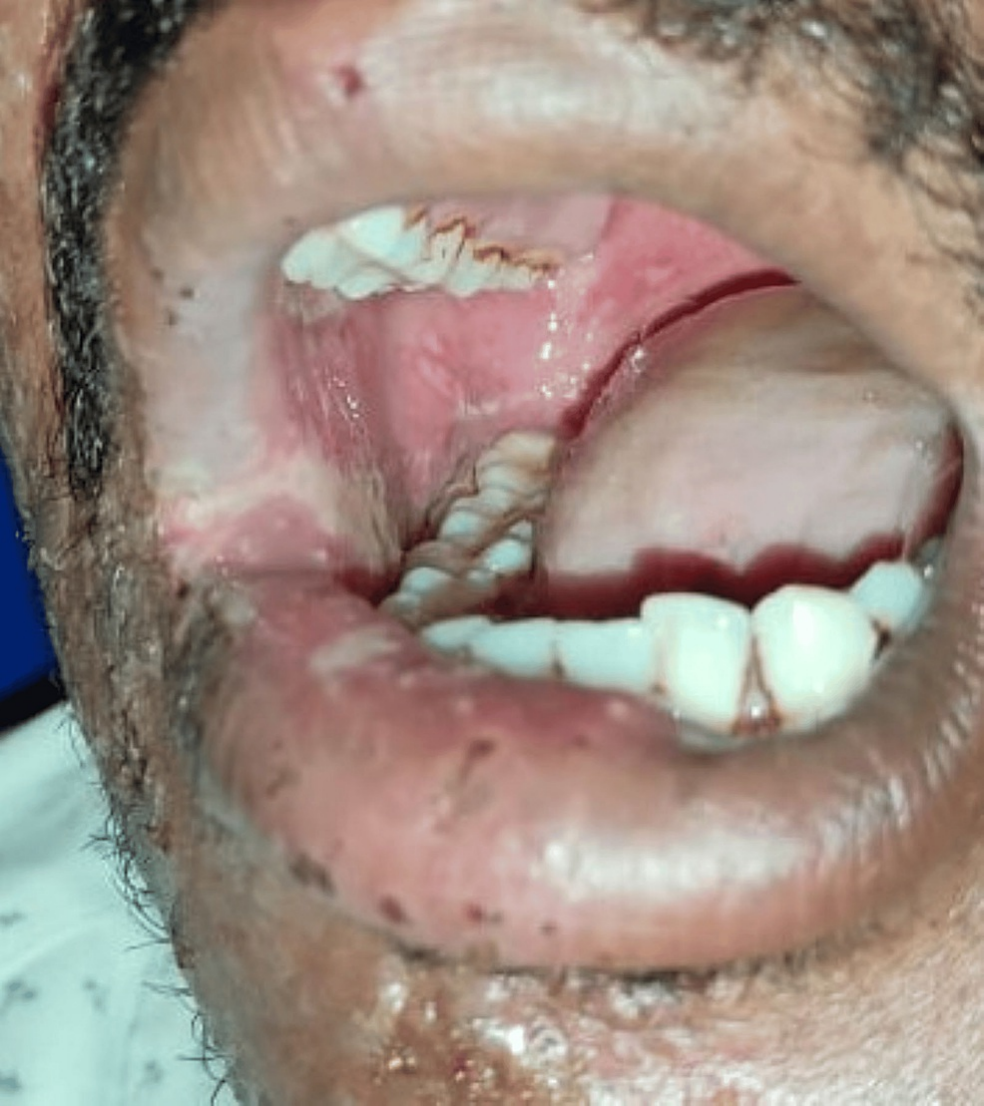
Multiple ulcers seen on the right buccal mucosa and the soft palate region in the oral cavity

**Figure 3:**
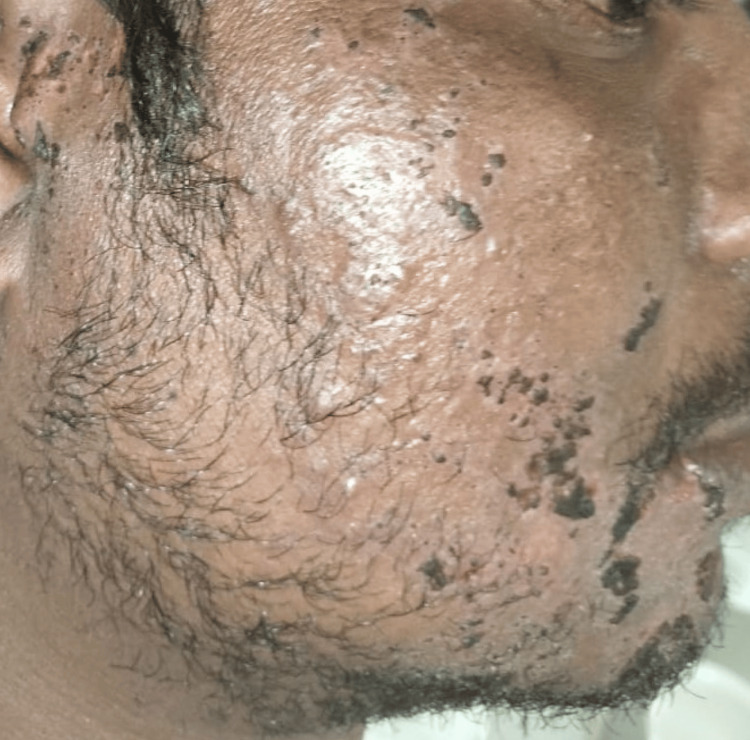
Healing of vesicles seen on the right region of the jaw and the auricular region

**Figure 4:**
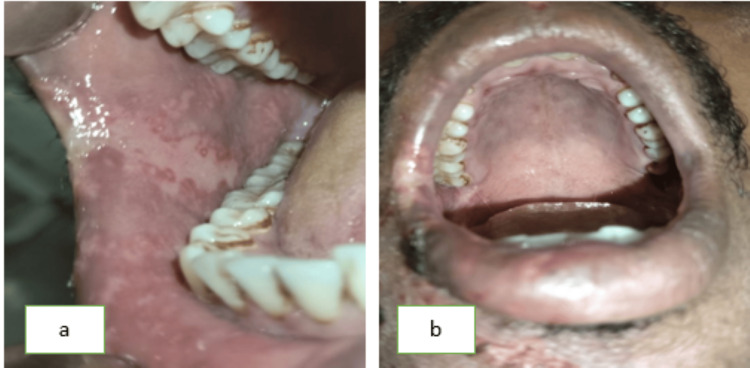
(a) Shows healing of ulcer with hypopigmentation on the right buccal mucosa. (b) shows healing of ulcer on the right region of the palate

A 36-year-old male reported to the Department of Oral Medicine and Radiology with chief complaints of severe pain and ulcer in the right upper and lower regions of the jaw for seven days. The pain was deep, aching, burning type, and continuous in nature. After 2-3 days of onset of these symptoms, the patient notices a blister on the right half of the face and within the oral cavity followed by ulceration. The patient also experienced difficulty in eating and swallowing. On extraoral examination, a pattern of unilateral, clustered, and linear distribution of vesicles, ulcers, and scabs with erythema was seen on the right half of the maxillary and mandibular regions of the jaw. Vesicles were also seen on the right auricular region (Figure 1). On intraoral examination, multiple ulcerations were seen on the right buccal mucosa and the right half of the palate region (Figure 2). Tenderness was present in these regions with a Visual Analogue Score (VAS) of 9 (Range from 1 to 10 with 1 being no pain and 10 being excruciating pain). Taking into consideration the patient’s history, pattern, and unilateral distribution of the lesion, a provisional diagnosis was given as HZ infection. After educating the patient and explaining the condition he is having, management of the lesion was initiated immediately by prescribing acyclovir tab 800 mg thrice a day for 10 days, acyclovir cream for local application thrice daily for 10 days, Haridrakhand powder twice a day for 10 days, and Triphala powder twice a day for 10 days to reduce inflammation. The patient was recalled after three days where significant improvement was seen. Pain and burning sensation have reduced drastically with a VAS score of 2. Healing vesicles and completely resolved erythema were seen extraorally (Figure 3). Intraorally, the healing of mucosal lesions was also seen (Figure 4). The patient was able to eat and swallow properly. The same medication was continued, and the patient was recalled after seven days where remarkable improvement was seen. The dosage of the medication was reduced to 500 mg for acyclovir tablet, and the frequency of topical application of acyclovir cream was reduced to twice a day, and both dosages of Haridrakhand and Triphala powder remained the same. The patient was on further regular follow-up for a period of one-and-a-half months with satisfactory results.

## Discussion

HZ infection when left untreated can lead to complications such as postherpetic neuralgia which is defined as pain that stays for 120 days [10]. Patients may experience debilitating pain which usually is stabbing, sharp, burning, or gnawing in nature which can last more than one month. Some patients unfortunately may experience this pain for years [11]. Immunocompromised patients usually experience a more severe varicella zoster infection (VZI) that may present very atypical, maybe bilateral, and affect multiple dermatomes. Immediate intervention and management of the condition are required to prevent such complications. HZ infection may also lead to a triad of facial paralysis ipsilaterally, otalgia, and vesiculations around the ear and auditory canal which is commonly referred to as Ramsay Hunt syndrome [11]. Almost all cases of HZ are treated with corticosteroids along with antiviral drugs to prevent postherpetic neuralgia. However, considering the side effects of corticosteroids, this patient was treated with a combination of antiviral drugs and herbal medications wherein the results are found to be satisfactory.

Haridra (*Circuma longa*) is an efficient ayurvedic medication with powerful antioxidant properties and contains vitamins C and E and Beta carotene. It is also effective in reducing inflammation and has an excellent effect in wound healing, and the effect of this property was seen in our case where the patient has remarkable improvement with inflammation and healing caused by the infection [12]. Similarly, Triphala is also a potent herbal medication which has a powerful anti-inflammatory property [13]. Although steroids are usually given to reduce inflammation, these herbal medications are equally effective in reducing signs and symptoms of inflammation as seen in this case report. The present case report suggests that it may be convenient to use these herbal therapeutics with little to no side effects to alleviate the symptoms of inflammation [14].

## Conclusions

In patients suffering from HZ infection, symptoms could be very daunting and intimidating, interfering with the lifestyle of the patient. Immediate treatment of the lesion is required to prevent further adverse complications. In this case, a combination of conventional treatment along with ayurvedic medication has been used where the patient has striking improvement with symptoms and lesions. This has proven that a meticulous combination of allopathic and herbal medications can improve the condition of patients with a significant reduction in possible side effects.
